# The Foreign Journals: Surgery

**Published:** 1842-01

**Authors:** 


### SURGERY.

### *On the Formation of an Artificial Anus.* By M. Amussat.

At the Académie de Médecine on the 7th of September, M. Amussat communicated two new operations for artificial anus, which he has performed since the last communication on the same subject in October, 1839. In these two cases the artificial anus was made in the right lumbar region, while in the first two it was made in the left lumbar region: but in all alike without opening the peritoneum. The first of the operations now described was performed on the 3d of July last, on a woman fifty years old. The intestinal canal had been completely obstructed for more than forty days: and now, two months and some days after the operation, the patient is in the most satisfactory state. The second was performed on the 21st of August, for an obstruction of forty-five days' standing, caused by a cancerous affection of the rectum. The patient died ten days afterwards of the progress of the cancer. M. Amussat then related the subsequent history of the two cases communicated in 1839. In the first the artificial anus was made by opening the left lumbar colon, without touching the peritoneum, in a woman forty-eight years old. The constipation was caused by an obstruction of the sigmoid flexure of the colon, and had existed twenty-six days: there was also severe stercoral tympanitis. The patient after having enjoyed pretty good health for a certain time died, five months after the operation, of peritonitis produced by the progress of the carcinomatous disease. In the second of these operations the artificial anus was also made on the left side, in a man sixty-two years old, for an intestinal obstruction consequent on cancer of the rectum. During the two years since the operation was performed, the condition of the patient has greatly improved, and there is reason to hope that the cancerous affection, already much retarded in its progress, will not yet for a long time compromise the success of the operation.

Lastly, M. Amussat communicated a third operation, in which he made an artificial anus last year in a woman forty-seven years old; but, by the method of Littre, opening the cæcum in the iliac fossa. The patient, who was in a state of extreme weakness, died in twenty-four hours, and at the autopsy a stricture (not suspected during life) was found at the angle of union between the transverse and the descending colon.

Reflecting on these operations (says M. Amussat) we see that with regard to the situation to be chosen, the artificial opening has been made in four cases without opening the peritoneum, twice on the left and twice on the right side, and on one other occasion by Littre's plan, on the cæcum. It is reasonable therefore that Callisen's operation, thus modified, should henceforth be performed as one less severe than that of Littre, and which is well calculated to prolong life.

*Bulletin de l'Académie Royale de Médecine. Octobre* 15, 1841.

---

### *On Abscesses in the Substance of the Walls of the Bladder.* By M. Civiale.

In these cases the pus may either be infiltrated in the cellular tissue, giving an increase of thickness to the bladder, or it may collect in layers between its membranes, or may be arranged in distinct abscesses varying in size and number. In most instances the abscesses form near the summit and the fore part of the bladder; not, as Chopart says, near the perineal portion. Their diagnosis is very difficult: there is seldom any external tumour, and when there is, its characters are too variable to decide whether there be matter, and whether it be without or in the walls of the bladder. Neither can the existence of an abscess be determined by the occasional discharge of pus; for that may happen when the bladder is sacculated. According to the surface of the bladder nearest which they collect, these abscesses discharge themselves either into the surrounding cellular tissue, or into the cavity. Their formation generally depends on the irritation of calculi, or on blows or wounds, but sometimes occurs without any appreciable cause. They are not always attended by pain, or by any peculiar disturbance of the functions of the bladder; nor is there any method of examination by which their existence can clearly be discerned. In numerous instances they have been either overlooked, or treated as cases of retention of urine, or imagined to exist when the bladder was only sacculated, or when the pus had collected in the tissue around it, as it does more commonly than in its walls.

[These are the results of M. Civiale's experience in several cases, which are referred to in the original paper.]

*Bulletin Général de Thérapeutique. Septembre*, 1841.

---

*On an Operation for the Cure of Myopia and the disposition to fatigue of the Eyes.* By M. Bonnet, of Lyons.

The author believes that certain forms of these affections depend on too great an energy of the muscles of the eye-ball, which, by pressing too forcibly on it, either elongate its axis, make the cornea prominent and produce myopia, or after a time render it painful and incapable of being longer used in the examination of small bodies. Believing this, he thought that the appropriate remedy would be found in removing some of the excessive pressure by dividing one or more of the muscles; and that chosen for the purpose was the inferior oblique. The results of ten cases in which the operation was performed are briefly given. In no case was it followed by any ill consequence; in all, with one exception, the distance of distinct vision for reading (after both inferior oblique muscles had been cut) was, on an average, nearly doubled, and that for seeing larger objects, such as for the recognition of features, was rendered three, four, or even five times greater than it was before. In all, moreover, with one exception in which there was only an improvement, the tendency to fatigue of the eyes after a moderate use of them, was completely removed.

*Gazette Médicale. Septembre* 4, 1841.

---

*Extirpation of the Submaxillary Gland.* By M. Colson, of Noyon.

It has been questioned, especially by M. Velpeau, whether this operation was really ever performed, or whether the supposed diseased salivary gland was not in each case merely a diseased submaxillary absorbent gland. In the present case the evidence of its having been truly the submaxillary gland which was removed seems nearly complete. The patient was 65 years old, and had had for eleven years a cancer of the lower lip which had been treated by a variety of caustics. At the time of the operation this was as big as a large nut and was ulcerated extensively; and there was besides, towards the right side of the base of the lower jaw and below it, a lobulated tumour, which was supposed to consist of enlarged absorbents. After removing the cancer of the lip, the author proceeded to this tumour, but was astonished to find that it was situated much more deeply than he had expected, and that, in the removal of it, it was necessary to divide the lingual branch of the fifth nerve, and the submental artery; and to expose the facial artery, the digastric, stylo-hyoid and mylo-hyoid muscles, and the hypo-glossal nerve. The operation was with much difficulty accomplished, and the tumour when removed was found to consist of the submaxillary gland (which was recognized by its being still inclosed in its fibrous capsule) and of a few enlarged, but not otherwise diseased, absorbents. The gland was converted throughout into encephaloid tissue, which was in parts softened and nearly fluid; it measured an inch and a half in one direction and about an inch in the other. The patient rapidly recovered from the operation, and eight months after remained perfectly well.

*Annales de la Chirurgie. Aout*, 1841.

*Cure of slight degrees of Squinting without Tenotomy.* By Dr. DIEFFENBACH.

WHEN the strabismus is but slight it often happens that after the division of one of the recti, its antagonist draws the eye too far in the opposite direction, and produces a strabismus only different in kind from that which existed before the operation. For these cases, therefore, Dieffenbach proposes, instead of dividing the muscles on the side *towards* which the eye squints, to cut out a portion of the conjunctiva from over the insertion of the muscle of the side *from* which it squints. The operation consists merely in raising up a fold of conjunctiva several lines wide with a pair of hooks, and cutting it off, with some of its subjacent cellular tissues, with a pair of curved scissors. The contraction of the cicatrix is sufficient to draw the eye into the straight position. In external strabismus, a larger portion of conjunctiva must be cut from over the internal rectus, than in cases of internal strabismus it is necessary to cut from over the opposite muscle; because the former kind of strabismus almost always depends on weakness, the latter on excessive energy of the rectus internus.

*Casper's Wochenschrift. September* 4, 1841.

---

*On the Treatment of Old Fractures by Division of Tendons.* By Dr. DIEFFENBACH.

DIEFFENBACH has several times, in old cases of fracture of the patella or the olecranon, where the portions were dragged far apart, divided the adjacent tendons so as to be able to bring the portions together and, by friction of them one upon the other, to excite such action as might end in the formation of a shorter and firmer bond of union. In some cases considerable benefit was obtained after all other means had failed; in others the result was negative. Two examples are detailed; in one, an old ununited fracture of the ulna, he divided the tendon of the triceps, fixed the upper portion of the bone in its right place by a bandage, and every fourteen days rubbed it well against the lower one: in three months the union was firm. In another example, an old distantly united fracture of the patella, he divided the ligamentum patellæ and the rectus femoris about three inches above the patella; then, by an appropriate bandage and constantly drawing the separated portions more closely together, he obtained at the end of some months a complete hardening of the interposed substance, and a considerable amelioration of the patient's state.

*Casper's Wochenschrift. October* 2, 1841.

---

*On the Contagious Properties of Gonorrhœal and Blennophthalmic Matter.* By Dr. DECONDÉ.

THE object of this memoir is to describe the experiments, of which the results were detailed in a former one, and by which it was proved that gonorrhœal matter would always produce purulent ophthalmia in dogs, unless it were applied very soon after the urethra had been washed by irritant injections, such as solutions of nitrate of silver.

The numerous experiments now briefly detailed were instituted on the eyes of dogs, and in many instances on those of the author himself. They afford very strong evidence, 1st, that fluid from a urethra into which nitrate of silver has on the same day been injected, will neither produce gonorrhœa in man, nor granular ophthalmia in man or dogs; 2d, that if the same kind of fluid be mixed with a small quantity of chloride of lime it loses the whole of its contagious property, and does not regain it even after the evaporation of all the chlorine from it; 3d, that it is just the same with the purulent fluid of contagious ophthalmia, so that if within a few seconds of the contact of some of it with the conjunctiva, a small quantity of chloride of lime in solution be inserted within the eyelids, the contagion is entirely prevented, which if the remedy be not almost immediately applied, is sure to manifest itself.

*Annales de la Société de Médecine d'Anvers.*

*L'Examinateur Médical. Octobre* 24, 1841.

*On a rare kind of Stony Concretions in the Skin.* By Dr. JULIUS VOGEL.

J. W., a baker, 33 years old, had always enjoyed good health. In his fourteenth year, without any obvious cause, he was affected with a severe itching in the scrotum, which lasted a long time. It gradually ceased, but there then formed in the scrotum a number of small nipple-like elevations, which gradually enlarged till they acquired the size of a pea or a hazel nut, and then dried and wasted; but had scarcely dried in one part of the scrotum before similar tumours appeared in other parts. Since his fifteenth year he has constantly suffered from these tumours, which dry up and appear again at irregular periods, but which have never given him so much annoyance as to lead him to seek to have them cured till now, when he wants to marry.

The scrotum is not enlarged, but is beset with a great number of round tumours of the sizes just mentioned. They cover the whole scrotum and are about 150 in number; the skin upon and around them has its ordinary colour and appears quite normal; they are situated in or immediately beneath the corium, and are freely moveable; they are tolerably firm and give no pain.

One of these tumours was punctured with a lancet and its contents were pressed out. The substance obtained was greasy, white, and of the consistence and aspect of clotted milk; it felt soft and was just like the contents of an atheroma. Under the microscope it appeared like a granular amorphous substance, of a brown colour, with numerous colourless portions of crystals which were for the most part rounded and had no definite form. It contained no organized structures. Chemically examined it was found to consist chiefly of phosphate and carbonate of lime, with a trace of a salt of soda, and a small quantity of fat and extractive matter.

To see whether the opinion of Voigtel (who has described a similar case) were correct, that these concretions are formed in the sebaceous glands, Professor Stromeyer (under whose care the patient was) cut one of the tumours out and gave it to the author to examine; but all the tissues were so encrusted with the earthy matter that he could obtain no definite result, though he saw enough to make him think Voigtel's view very probable.

Neither the cause, nor the mode of production, nor the best mode of treatment of this disease, are at present more than obscurely known.

*Allgemeine Zeitung für Chirurge innere Heilkunde und ihrer Hülfswissenschaften.* Nr. 1. *Juli* 10, 1841.

---

*Fatal Stab of the Brain.* By Dr. KEYL, Regimental Surgeon.

THE chief interest of this case which is given at great length in the Public Reports, lies in the absence of immediate symptoms in consequence of a severe injury of the head. A drummer was stabbed in the left temporal fossa, with a common clasp-knife. The wound bled a good deal, but after washing it with the help of his comrade, he went home to his quarters and slept well all night. Next day he remained very well, and in the afternoon at the hospital, he complained of only a slight pain in the wound, which, as it did not seem of much importance, was covered with simple dressing. On the day following he still remained perfectly well in his intellects and in his general health; and it was not till the sixth day after he received the wound, that symptoms of inflammation of the brain set in. Of these, in six days more he died.

On examination it was found that the knife, after traversing the temporal fossa from above downwards, and from before backwards, had passed obliquely through the squamous portion of the temporal bone, (in such a manner that it could not be discerned during life till the wound was dilated,) and had then penetrated to an equal extent the dura mater, the inferior part of the posterior lobe of the cerebrum, the tentorium cerebelli, and the left lobe of the cerebellum in which it had passed as far as the arbor vitæ. All these parts now showed signs of having been acutely inflamed.

*Medicinishe Zeitung. Juli* 21, 1841.

*Researches on Puncture of the Bladder.* By M. MONDIERE.

IN this paper the author cites 92 cases of puncture of the bladder, the results of which may be arranged in the following manner:

| | Puncture | | | |
|---|---|---|---|---|
| | perineal | recto-vesical | hypogastric | Total |
| Number of cases | 9 | 28 | 55 | 92 |
| Success | 6 | 19 | 49 | 74 |
| Fistula | 1 | 3 | 0 | 4 |
| Infiltration | 0 | 3 | 0 | 3 |
| Abscess | 0 | 1 | 0 | 1 |
| Hemorrhage | 1 | 0 | 0 | 1 |
| Death | 1 | 2 | 6 | 9 |

*Gazette Médicale de Paris.* 21 *Aout,* 1841.
*From Revue Médicale. Avril,* 1841.

---

*On Paralysis of the Muscles of one side of the Face, and Division of those of the opposite side.* By Dr. DIEFFENBACH.

IN two cases met with many years ago, the author, to remedy the extreme deformity of paralysis of one side of the face that had existed many years, cut a considerable portion of skin out of the cheek of the paralysed side, and stitching the edges of the wound together, contracted the part and considerably improved the patient's appearance. However he was not satisfied with this operation, and finding that in all such cases the unresisted healthy muscles became rigid, like some of those of the leg, in paralytic club-foot, he determined on dividing them by subcutaneous incision. He made his first essay in an old case of paralysis of the orbicularis palpebrarum; he divided the levator palpebræ by passing a very narrow knife under the skin of the upper eyelid and cutting downwards on a piece of wood put between the lid and the globe ; the result was striking, for the eyelid instantly closed and opened again ; it closed because the paralysis was not complete ; it opened because some fibres of the levator had intentionally been left undivided. In three other cases the result was equally remarkable and successful; and in none of these did any serious effect follow ; but in a fifth case the eyelid became acutely inflamed, ulcerated, was perforated, and required an autoplastic operation to remedy the mischief. In a number of cases more, Dieffenbach has since divided all the muscles of one side of the face (in the same manner as he does those which in another class of cases are spasmodically convulsed), and in a great majority of them the operation was more successful than could have been expected. The contracted unresisted muscles were instantly so enervated that the overpowered ones of the other side at once began to act again; and even in the very oldest and worst cases, where the paralysed cheek was completely atrophied and hung down in a pouch, a considerable improvement of appearance was obtained.

*Medicinische Zeitung. September* 15, 1841.

---

*On the Application of the Subcutaneous Method to the opening of Chronic Abscesses.* By M. JULES GUERIN.

IT is generally admitted that the fatal termination of psoas and other abscesses of the like kind is due to the effects of the admission of air into their cavities, and various, but for the most part insufficient, methods have been proposed at different times to prevent that accident. M. Guerin's plan is as follows: he uses a flat trocar, long, but of small diameter, and enclosed tightly in a canula. The canula is furnished with a cock near its larger end, which, when open, permits the trocar to pass, but, when shut, exactly closes the canula. The outer end of the canula fits on the nozzle of an ordinary syringe, and all those parts are so adapted that no air may pass where they fit one to the other. A fold of skin being made at some little distance from the abscess, the trocar and canula are introduced at its base, and carried under the integuments till the point of the former has entered the sac; then the trocar is slowly withdrawn (an assistant gently pressing the walls of the abscess,) and at the instant that its point passes the cock, *that* is turned and the pus prevented from flowing out. The syringe is now adapted, the cock turned back, and the pus sucked out of the sac at once, or with one or more emptyings and refillings of the syringe, if its quantity be very large. When this is finished the canula is slowly withdrawn, care being taken to keep the walls of the track through which it passes close together. A pad and bandage are then put on the sac, and the track leading to it, and the orifice of the latter is closed with sticking plaster.

Such is an outline of M. Guerin's plan, to which he has added lengthened details, most of which will at once suggest themselves to an ingenious operator. His experience of its effects are, that he has employed it "eleven times in opening abscesses by congestion, the consequences of tubercular affections of the bones, and eleven times in opening cold abscesses properly so called, and in no case has he observed any local or general accident that could be referred to the operation." It is not pretended that the method cures the diseases on which the abscesses depend, but only that it obtains all the advantages of evacuating the pus without any of the dangers of the older operations.

*L'Examinateur Médical. Septembre* 19 *et* 26, 1841.

---

*On the Curative Influence of Galvanism in some Organic Diseases of the Eye.* By Drs. Lerche and Kabat.

In the last number some account was given of this mode of treatment: the two papers before us contain notices of seven more cases of cataract in which it has been tried. The result appears on the whole unsatisfactory. The common effect of the electro-galvanic action is to produce during the operation considerable pain, and subsequently severe and sometimes very obstinate inflammation of all the tissues of the eye. In the course of this the cataract has been in some cases partially absorbed, and the sight in a measure improved; but the attainment of these, the most favorable of its results, cannot be relied on, and in some of the cases the operation manifestly did harm. Dr. Lerche's conclusion, which is of course drawn with some partiality for the plan, is, "that it is an important remedy in some organic diseases of the eye, but that its application requires great caution, and must be confined to those cases of cataract in which a favorable result is scarcely to be hoped for from the common modes of operation."

*Medicinische Zeitung. September* 1 *&* 8, 1841.

---

*On the Cure of Obliquity of the Nose by Subcutaneous Division of the Cartilage.* By Dr. Dieffenbach.

This condition of the nose which, however common in a slight degree, is, in an extreme, remarkably ugly, has been twice cured by Dieffenbach. The patients were young persons about 20 years old. In one the obliquity was congenital, in the other the consequence of a fall; in one the nose was turned to the right, in the other to the left, and in both to such an extent that it was thrust completely out of the middle of the face with its point turned towards the cheek, and the nostrils lying almost one over the other. The operator ran a small knife through the skin on the side of the bridge of the nose where the cartilaginous and osseous parts meet, and carried his incision so as to separate from the bone the bridge of the nose, one of its lateral walls, and a flap beneath the skin. Then through another puncture on the other side just under the bridge, he divided in the same direction the cartilage and the septum. The nose could now be without difficulty put right; compresses kept it so; no ill consequences followed; and shortly the patients were both so much improved that they could barely be recognized.

*Casper's Wochenschrift. September* 18, 1801.

*On the Effects of the Absolute Repose of Joints, without previous Disease.*
By M. TEISSIER, of Lyons.

TEMPORARY or permanent stiffness of a joint, it is well known, is a frequent consequence of its being maintained for a length of time in the same position. The chief object of the author is to prove by examinations of the dead body, that the morbid changes produced are more serious than is generally supposed. They are, he says, besides the muscular rigidity which always follows rest, effusion of serum and blood into the articular cavities, vascular injection of the synovial membranes, formation of false membranes, alterations, such as softening, roughness, and absorption of the cartilages without adhesion of the articular surfaces, and sometimes complete anchylosis. Of all these changes he has seen examples in the limbs of those who have been long confined with fractures; and this not only in the joints near the seat of fracture but in those of the same limb most distant from it; for instance, in the tarsal and metatarsal joints of limbs in which the femur had been broken. In nearly all cases the changes take place without pain or any other symptom of disease.

[The morbid changes which the author describes do not seem referrible to any other cause than that to which he ascribes them, however improbable it may seem that mere rest of a joint should give rise to such changes as are commonly produced by inflammation. M. Ricord and M. Cloquet have seen similar changes in some cases of complete local paralysis.]

*Gazette Médicale. Sept.* 25 *et Oct.* 2, 1841.

---

*Case of Fistula of the Larynx, with complete Closure of the Tube above it.*
By M. REYNAUD, of Toulon.

A MAN, twenty-eight years old, cut his throat so as to divide the anterior surface of the pharynx, the posterior and lateral parts of the cricoid cartilage just below the inferior cornua of the thyroid, the crico-thyroid membrane, and all the soft parts in front of it. As the wound healed his breathing became gradually more and more difficult, and to relieve himself he cut all the same parts again to a somewhat less extent. As they again healed the same dyspnœa returned, and now he wore a tube in the trachea; but one day the end of it fell into the bronchus, and being taken into the hospital for its extraction he came under the observation of the author, who found that the larynx above the fistula was so completely closed that neither water nor mercury poured through the glottis came down to the fistulous opening. After death, which was produced by repeated attacks of bronchitis, it was found that the integuments which had fallen in at the upper part of the wound, and the pharynx which had been drawn forward, were united so as to form a complete oblique partition just below the thyroid cartilage. The glottis was small, but healthy.

The most singular fact in this case was that, though he had lost his voice, the man could speak with sufficient distinctness to be plainly understood at a distance of four feet. He had great difficulty in pronouncing the letters *a, e, l;* still more in pronouncing *o;* and *m* and *n* were quite impracticable. When he wanted to speak he opened the mouth, depressed the pharynx, and after having filled the vocal tube with air, suddenly elevated the larynx and spoke by jerks, as if he were expectorating, always leaving a little interval before the pronunciation of each word. His pronunciation, though distinct enough to be understood, was not clear; nor could he, without much fatigue, maintain a long conversation. He could whistle a little, blow his nose, spit, and sneeze; but all these he did with difficulty, by a sudden and violent elevation of the larynx, after having inspired deeply.

[The details of this case, even in the longer memoir from which the above abstract is made, are too imperfect to teach all that might have been learnt from them; it is not even mentioned what sound was made in speaking, whether it were a mere whisper or more. The impossibility or difficulty of sounding the letters mentioned, renders it nearly certain that no air was ever made to pass through the glottis. It is probable that the violent elevation of the larynx, and with it the thrusting forward of the upper part of the pharynx and the fauces, were sufficient to impel the air in the mouth with force enough to be formed into the sounds of the different letters by the tongue, teeth, and lips. The chief result of the case therefore is to show for what articulate sounds the glottis is necessary.]

*Gazette Médicale. Septembre* 10, 1841.

---

*On Division of the Muscles of the Face when affected with Chronic Spasms.* By Dr. Dieffenbach.

Three cases (in addition to one detailed in our review of the author's work in the present Number, p. 22), have been operated on, and in all the results were completely successful. The disease had in all the patients existed for several years, and affected in all a number of the muscles of one side of the face, so that the eye was often violently closed, and the angle of the mouth involuntarily pulled up or downwards. To divide the orbicularis palpebrarum sufficiently, three subcutaneous incisions were necessary, one upwards, a second downwards, and a third outwards. They were all made with a fine curved knife, run in at the level of the outermost fibres, carried to the edge of the tarsal cartilage, and in withdrawing it made to cut through the whole breadth and thickness of the muscle, and down upon a thin layer of wood with which the eye-ball was defended. The section through the cheek extended from the ala of the nose to the anterior border of the masseter; the knife being passed in at the latter place and carried with its back just under the skin to the former. To divide the muscles of the angle of the mouth, a similar section was made from it obliquely downwards to the lower edge of the lower jaw.

The immediate result of the operation was cessation of the spasmodic contractions, either complete, or so as to leave but a slight vibration. Pads of charpie strapped down pretty firmly were necessary to limit the hemorrhage under the skin, but no severe symptoms ever followed, and within a few days every one of the patients was thoroughly well.

*Medicinische Zeitung. September* 15, 1841.

---

*On a New Mode of Treating Tinea Favosa.* By M. Petel, of Louviers.

The treatment of the cases of tinea in the Hôpital des Enfans Malades at Paris is intrusted to the Messrs. Mahon, who have obtained great success by the employment of some secret remedy.

M. Petel's object has been to arrive by some rational mode at results similar to those obtained by the Messrs. Mahon, whose secret remedy, in common with many other nostrums, has the effect of producing the separation of the hair; an effect usually regarded as indispensable to the cure of tinea. The grand aim was to arrive at the knowledge of some good depilatory process, and in imitation of MM. Mahon, M. Petel has composed an ointment and a powder.

The ointment consists of

| | | | | Avoirdupois. |
|---|---|---|---|---|
| Common soda . . . . | 60 | centigrammes | = | about 9 grains. |
| Slacked lime . . . . | 4 | grammes | = | „ ʒj. |
| Lard . . . . . . . . . | 120 | „ | = | „ ℥ij. |

The powder is formed of

| | | | | |
|---|---|---|---|---|
| Quick lime . . . . . | 120 | „ | = | „ ℥ij. |
| Powdered charcoal . . . | 8 | „ | = | „ ʒij. |

When a person applies who is affected with tinea, M. Petel orders the hair to be cut about a quarter of an inch from the skin; he then gets rid of the crusts by poultices, and frequent washings with soap and water. At about the sixth day he commences the employment of the ointment which is repeated daily, the head being kept clean by means of a fine comb, which should be greased, and by frequently washing it with soap and water. The reproduction of the favous crusts gradually ceases, though frequently six weeks are required for that purpose; and when that object has been accomplished M. Petel sprinkles some of the powder among the hair every other day. The hairs by degrees lose their adherence to the scalp and may be pulled out without putting the patient to any pain.

When all the hairs have been removed nothing more is requisite than to apply the ointment daily until the hair has regained its natural colour.

M. Petel says that this mode of treament has been followed by great success; it seems, however, far inferior to the plan recently adopted by M. Devergie, at the Hôpital St. Louis, of applying certain caustics to the favi. A solution of acid nitrate of mercury of the Paris pharmacopœia was applied with a brush to the favi in a very aggravated case, in which the disease occupied the posterior part of the head. The favi immediately assumed a reddish-yellow colour; they fell off on the fifth day, leaving healthy skin beneath, and did not reappear.

A caustic solution of iodine was employed with similar success in two other cases. The observations of M. Gruby, with reference to the vegetable nature of the favi, led to a trial of these applications, which need to be employed more extensively in order to confirm the favorable results obtained from their use in a few cases.

*Bulletin Général de Thérapeutique. Octobre,* 1841.

---

### *On Strangulated Herniæ.* By M. MALGAIGNE.

THE object of this long memoir is to prove that the condition upon which the symptoms depend, and to which the treatment must be directed, in what is called strangulated hernia, is in many, and perhaps in the majority of cases, a simple inflammation of the sac and its contents. This, the author holds, is especially true in large and old intestinal herniæ, which have never been kept up by a truss, or where the truss has been long left off; in these there is never any strangulation, for the ring or rings are always larger than the pedicle of the hernia requires. In pure epiploceles also, he says, strangulation has never yet been proved to exist; their being irreducible depends on an adhesive or suppurative peritonitis having taken place, and giving rise to the symptoms. In both these classes of cases the operation is irrational and mischievous; and in general, the advice which the author urges is a delay of the operation (unless there be almost certain evidence of strangulation) till the measures calculated to produce inflammation have been employed. These, in some cases, remove the symptoms, though the hernia may itself appear unaffected; in many others they render reduction easy, which was before impracticable.

These conclusions are supported by the recital of some striking cases, and by references to a great number more. Enough is proved to render it certain that, in many cases which would commonly be operated on, the liberal application of leeches, cold, and other treatment adapted to the peritonitis, may prove sufficient for their perfect cure; and that in all, the inflamed state of the sac and its contents deserves more attention than it commonly receives.

*Archives Générales de Médecine. Octobre et Novembre,* 1841.

---

### *Statistical Researches on Dislocations.* By M. MALGAIGNE.

THE results in this memoir (which is supplemental to one of the same kind on fractures), are drawn from an examination of the records of the Hôtel Dieu during a period of sixteen years, in which 530 dislocations were entered as seen or admitted. Although the records were not always carefully kept, yet there is sufficient harmony in the general results to render it probable that they are true. The following are the most interesting among them:

Dislocations are one fourth more numerous in the first and last quarters of the year than in the second and third; and their increased number is always in cold weather in a greater proportion than that of fractures is. In relation to age, dislocations are very rare in early childhood, thence, gradually become more frequent to the 15th year; thereafter rapidly increase in frequency, and then again slowly become more common up to the age of 65, after which they seemingly become more rare. But, taking the proportion of those who suffer dislocations to the whole number of the population, it becomes regularly and considerably greater from the 15th year to the end of life; and in comparison with other ages, old age is less subject to fracture than to dislocation; in other words, as age increases, the liability to dislocation increases more rapidly than the liability to fracture. In young people dislocations, like fractures, are more common in summer that in winter; in old people the reverse holds. Men are three times more liable to them than women are. The limbs of the right side are more often dislocated than those of the left, in the proportion of five to four. Of the whole number of dislocations those of the humerus constitute at least two thirds; those of the lower extremities one eighth; and the proportion of the dislocations of the humerus to the whole number, regularly increase with advancing years, so that whilst it is only a quarter before the age of 15, it is four fifths after that of 55, and six sevenths after 70. Next to dislocations of the humerus those of the clavicle are most frequent, and they are especially so in manhood: then come the dislocations of the femur, and then those of the elbow, both of which also are most common between the ages of 25 and 40. The number of fractures that occur is altogether about six times as many as that of dislocations; but fractures of the humerus are very little more frequent than dislocations of it, while fractures of the femur are more than as fifteen to one case of dislocation, those of the clavicle as nine to one, of the fore-arm as twelve to one, and of the leg as 100 to one.

*Annales de la Chirurgie. Octobre,* 1841.

